# Coordinated transcriptional regulation of two key genes in the lignin branch pathway - *CAD *and *CCR *- is mediated through MYB- binding sites

**DOI:** 10.1186/1471-2229-10-130

**Published:** 2010-06-28

**Authors:** Anjanirina Rahantamalala, Philippe Rech, Yves Martinez, Nicole Chaubet-Gigot, Jacqueline Grima-Pettenati, Valérie Pacquit

**Affiliations:** 1 Université de Toulouse; UPS; UMR 5546, Surfaces Cellulaires et Signalisation chez les Végétaux; BP 42617, F-31326, Castanet-Tolosan, France; 2 CNRS; UMR 5546; BP 42617, F-31326, Castanet-Tolosan, France; 3 Université Pierre et Marie Curie Paris 6, EAC7180 CNRS, UR5, Mécanismes de la Régénération des Plantes, F-75252 Paris cedex 05, France

## Abstract

**Background:**

Cinnamoyl CoA reductase (CCR) and cinnamyl alcohol dehydrogenase (CAD) catalyze the final steps in the biosynthesis of monolignols, the monomeric units of the phenolic lignin polymers which confer rigidity, imperviousness and resistance to biodegradation to cell walls. We have previously shown that the *Eucalyptus gunnii CCR *and *CAD2 *promoters direct similar expression patterns in vascular tissues suggesting that monolignol production is controlled, at least in part, by the coordinated transcriptional regulation of these two genes. Although consensus motifs for MYB transcription factors occur in most gene promoters of the whole phenylpropanoid pathway, functional evidence for their contribution to promoter activity has only been demonstrated for a few of them. Here, in the lignin-specific branch, we studied the functional role of MY*B *elements as well as other *cis*-elements identified in the regulatory regions of *EgCAD2 *and *EgCCR *promoters, in the transcriptional activity of these gene promoters.

**Results:**

By using promoter deletion analysis and *in vivo *footprinting, we identified an 80 bp regulatory region in the *Eucalyptus gunnii EgCAD2 *promoter that contains two MYB elements, each arranged in a distinct module with newly identified *cis*-elements. A directed mutagenesis approach was used to introduce block mutations in all putative *cis*-elements of the *EgCAD2 *promoter and in those of the 50 bp regulatory region previously delineated in the *EgCCR *promoter. We showed that the conserved MYB elements in *EgCAD2 *and *EgCCR *promoters are crucial both for the formation of DNA-protein complexes in EMSA experiments and for the transcriptional activation of *EgCAD2 *and *EgCCR *promoters in vascular tissues *in planta*. In addition, a new regulatory *cis*-element that modulates the balance between two DNA-protein complexes *in vitro *was found to be important for *EgCAD2 *expression in the cambial zone.

**Conclusions:**

Our assignment of functional roles to the identified *cis*-elements clearly demonstrates the importance of MYB *cis*-elements in the transcriptional regulation of two genes of the lignin-specific pathway and support the hypothesis that MYB elements serve as a common means for the coordinated regulation of genes in the entire lignin biosynthetic pathway.

## Background

Vascular cambium is a cylindrical secondary meristem that produces both secondary phloem and secondary xylem (*i.e. *wood in trees). The most characteristic components of secondary cell walls are lignins, complex phenolic polymers, which play fundamental roles in mechanical support, water and solute conductive properties and disease resistance in higher plants [1]. The biosynthesis of the lignin polymers derives from the general phenylpropanoid pathway which provides precursors for several branch pathways leading to the elaboration of a wide range of compounds involved in various aspects of plant development and defence [2]. In the lignin-specific branch pathway, the conversion of hydroxycinnamoyl CoA esters into cinnamyl alcohols (or monolignols), the monomeric units that are incorporated into the lignin heteropolymer, is catalyzed by cinnamoyl CoA reductase (CCR; EC 1.2.1.44) and cinnamyl alcohol dehydrogenase (CAD; EC 1.1.1.195) successively.

The spatial and temporal expression of the *Eucalyptus gunnii CCR *and *CAD2 *genes was investigated by fusing the gene promoters to the *uidA *reporter gene coding for *β*-glucuronidase (GUS) and expressing these constructs in transgenic woody and herbaceous plants [3-6]. Both *EgCAD2 *and *EgCCR *gene promoters have been reported to direct GUS activity in the vascular tissues of all organs in all the plant species examined. Similar expression patterns have been reported for the *Arabidopsis thaliana AtCAD-C *and *AtCAD-D *genes, which encode proteins that are closely related to the *Eg*CAD2 enzyme [7]. The GUS activities were found to be consistent with tissue and cell locations of the *CCR *and *CAD2 *transcripts and proteins obtained by the use of *in situ *hybridization and immunolocalization [8,9]. These observations showed that the two genes have the same expression pattern, suggesting that the control of monolignol production is, at least in part, achieved through their coordinated transcriptional regulation.

Deletion studies of the *EgCCR *and *EgCAD2 *promoters showed that the *EgCCR *promoter deleted to -119 bp upstream of the transcription start site retained its ability to direct GUS expression in a pattern similar to that obtained with the full-length promoter whereas the promoter deleted to -70 bp was inactive [5]. In the *EgCAD2 *promoter, a region necessary for GUS expression in cambium and secondary xylem was mapped between -340 and -124 bp upstream of the transcription start site [6]. These results indicate that expression of the *EgCCR *and *EgCAD2 *genes is under the control of a promoter region (50 bp in *EgCCR *and 216 bp in *EgCAD2*) proximal to the transcription start site. As revealed by *in silico *studies, both regulatory regions contain putative binding sites for MYB transcription factors.

Plant MYB proteins belong to one of the largest families of transcription factors. According to their predicted sequences and structural features, they have been classified into several subgroups [10-12]. The R2R3 two-repeat MYB family occurs specifically in plant lineages and its members have been postulated to participate in the regulation of a wide range of developmental and metabolic processes, notably the phenylpropanoid biosynthetic pathway [13-15]. However, despite the conservation of MYB consensus motifs (previously called AC-elements) in most phenylpropanoid gene promoters, functional evidence for their contribution to the promoter activity has only been directly proven for a few of them (reviewed in [16]). Although the involvement of MYB proteins in the monolignol-specific branch pathway has been suggested (reviewed in [17]), the functional role of MYB *cis*-elements and the molecular mechanism by which they participate in promoter activities have not yet been investigated.

The main goal of this study was to define the functional role of putative *cis*-elements in the transcriptional activities of *EgCAD2 *and *EgCCR *promoters. Firstly, we restricted the regulatory region of the *EgCAD2 *promoter to an 80 bp region necessary for expression both in secondary xylem and in cambium. This 80 bp region contains two MYB sites and two unreferenced sites that are revealed by *in vivo *footprinting. A straightforward approach to investigate the functional role of promoter elements is to introduce block mutations into the putative *cis*-elements in the context of the shortest active promoter. Thus, by using *EgCCR *and *EgCAD2 *short promoter versions carrying single or combined mutations in their putative *cis*-elements, we clearly identified the MYB elements in the *EgCCR *and *EgCAD2 *promoters as being crucial not only for the formation of protein-DNA complexes *in vitro *either with recombinant MYB protein or with cellular protein extracts, but also for the transcriptional regulation of *EgCCR *and *EgCAD2 *genes *in planta*. In addition, the experimental data showed that a newly identified *cis*-element is required for *EgCAD2 *expression in the cambial zone.

## Results

### An 80 bp *EgCAD2 cis-*regulatory region is involved in *EgCAD2 *expression in xylem tissues and in dividing cells

**Figure 1:**
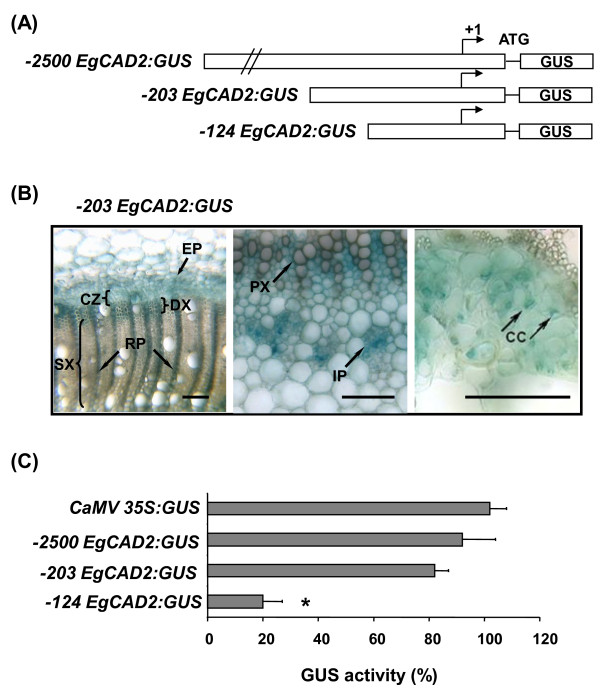
**Delineation of the regulatory region in the *EgCAD2 *promoter**. **(A) **Schematic maps of the 5'-deleted *EgCAD2 *promoter-*GUS *fusions. (+ 1), transcription start site; GUS, *uidA *coding region. (**B) **Histochemical analysis of GUS expression driven by the -203 *EgCAD2 *promoter in stem cross sections of transgenic tobacco plants. From left to right, general view of the secondary xylem zone, enlargement of primary xylem zone, enlargement of internal phloem. Bars represent 100 μm. CC, companion cells; CZ, cambial zone; DX, differentiating xylem; EP, external phloem; IP, internal ploem; PX, primary xylem; RP, xylem ray parenchyma; SX, secondary xylem. These images are representative of 12-17 greenhouse-grown, 8-week-old independent transformants.** (C) **Fluorimetric assays of GUS activity in transgenic tobacco BY2 cell calli. Histograms represent mean values and standard deviations of three measurements, each on 45 independent calli, expressed as % of the *CaMV 35S *promoter activity. Activity driven by *EgCAD2 *5'deletion promoter construct statistically different relative to the control (-2500 *EgCAD2) *is highlighted with an asterisk, (Student's *P *= 0.02).

To further localize the *cis*-elements in the 216 bp region ([-340/-124]) involved in the transcriptional regulation of *EgCAD2 *[6], we introduced several truncated versions of the promoter fused to the *GUS *reporter gene into tobacco by *Agrobacterium*-mediated transformation. Tobacco, a model herbaceous plant which undergoes secondary thickening, was previously shown to be a suitable model plant for studying the *EgCAD2 *promoter from trees [6] and also phenylpropanoid promoters from herbaceous or woody plants (see for example [18-20]). The shortest tested 5'**-**deletion showing a similar vascular expression pattern to the full-length (-2500 bp) promoter was deletion -203 (Fig. 1a). Stem cross-sections showed GUS expression in cells connected to lignification, such as differentiating xylem and parenchyma cells of the primary and secondary xylem (rays), but also in cells apparently unconcerned by lignification, such as the dividing cells of the vascular cambium and the cells of the external and internal phloems, especially the companion cells (Fig. 1b). GUS expression in xylem and cambium was lost upon deletion of the promoter to -124 which retained GUS activity only in the internal phloem of tobacco stem [6], delimiting a shorter (80 bp) regulatory region [-203/-124] involved in promoter activity in xylem and vascular cambium.

To confirm the importance of this 80 bp region in *EgCAD2 *expression in dividing cells, we transformed tobacco BY2 suspension-cultured cells with the -203 promoter deletion construct. Fluorimetric measurements of GUS activity indicated that this short promoter was active in BY2 cells at a level close to that of the *EgCAD2 *full-length promoter or of the constitutive *CaMV 35S *promoter used as a reference (Fig. 1c). GUS activity decreased drastically with the -124 promoter deletion construct (Fig. 1c). These data confirm that the [-203/-124] region contains *cis*-elements required for activity of the promoter in dividing cells (cultivated *in vitro *and meristematic cambial cells) as well as in xylem.

### Fine mapping of *cis*-elements in the *EgCAD2 *promoter by *in vivo *DMS footprinting

To precisely map the *cis*-elements in the *EgCAD2 *promoter interacting with *trans*-acting factors, we used *in vivo *dimethyl sulphate (DMS) footprinting, a technique allowing location of protein-DNA interactions with single nucleotide resolution [21]. The chemical agent DMS methylates the unprotected guanine residues rendering them susceptible to cleavage by piperidine.

**Figure 2:**
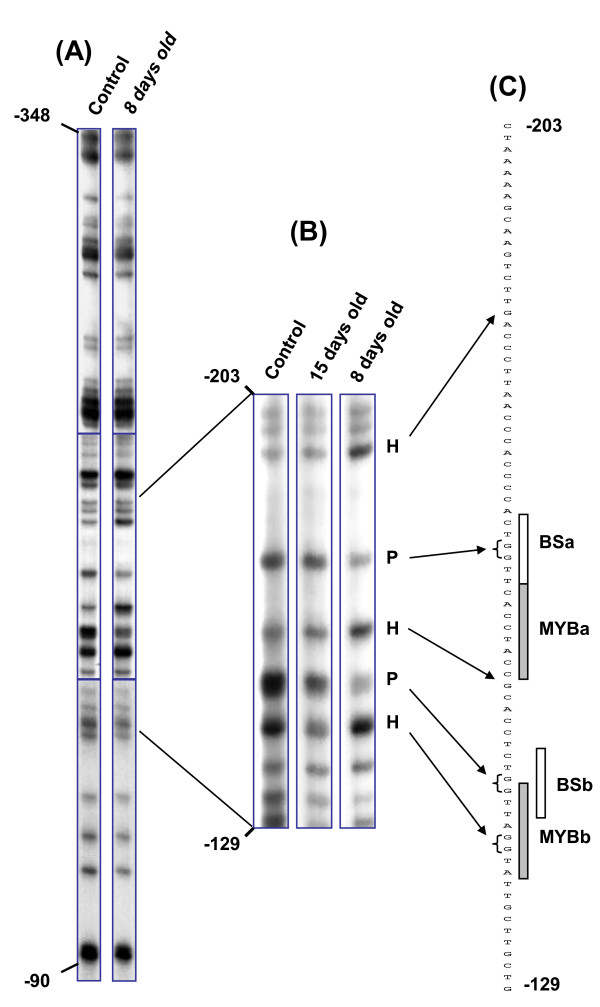
***In vivo *footprinting of the *EgCAD2 *promoter**. (**A**) DMS footprint profiles were investigated in the -348/-90 promoter region. (**B**) Enlargement of the -203/-129 region. The protected (P) or hypersensitive (H) residues in exponentially growing *Eucalyptus *suspension-cultured cells (8-day-old) and stationary phase cells (15-day-old) are revealed by comparison with *in vitro*-treated genomic DNA (control). (**C**) Sequence of the regulatory region. Grey boxes correspond to the MYB sites (MYBa and MYBb) and white boxes correspond to BSa and BSb sites (see text). Representative footprints of eight independent experiments are shown.

The footprints were examined over a broad region [-348/-90] encompassing the regulatory region (Fig. 2a). In exponentially-dividing cells that highly expressed *EgCAD2 *(Day 8, see these data in additional file 1), several guanine residues of the promoter sequence showed differences in the footprint relative to the control (protein-free DNA), whereas in stationary phase cells with low expression of *EgCAD2 *(Day 15) the footprint was almost identical to the control (Fig. 2b). The protected (P) or hypersensitive (H) sites, corresponding to lower or higher intensity bands respectively relative to the control, are indicated on the promoter sequence (Fig. 2c). Two protected areas were identified, each composed of two adjacent guanines (positions -166, -167 and positions -146, -147), surrounded by hypersensitive residues (positions -141, -142, -155, -187). Hypersensitivity to DMS methylation can indeed result from alterations in the local DNA topology induced by protein binding [21]. The protected guanines fall within two identical sequences (CTGGTT), which we call BSa (binding site a) and BSb (Fig. 2c), and which have no homology with consensus binding sequences in databases. Just downstream of both BSa and BSb, however, are sequences that correspond to MBSIIG, one of the plant MYB transcription factor binding sites (consensus motif G(G/T)T(A/T)GGT(A/G); [22]). These elements are named MYBa (-163 -156 in reverse orientation) and MYBb (-146 -139 in direct orientation). All the footprints are included within the 80 bp regulatory region delineated above. No other footprint was observed within the investigated [-348/-90] broad promoter region (Fig. 2a), indicating the absence of additional interaction sites upstream and downstream of the 80 bp regulatory region.

When surveying the *CAD2 *regulatory region in several *Eucalyptus *species, a few base substitutions were found in BSa and BSb sequences whereas the MYB sites were perfectly conserved, arguing for their possible functional importance (see additional file 2). It is worthy of note that similar conservation across *Eucalyptus *species was also found for the unique MYB site in the *CCR *regulatory region (additional file 2).

These results suggest that four relatively close putative *cis*-elements in the regulatory region [-203/-129] of the *EgCAD2 *promoter could interact with several transcription factors in cells that are transcriptionnally active for the *EgCAD2 *gene.

### Effect of mutations on *in vitro *and *in vivo *binding of *Eg*MYB2 to the *EgCAD2 *and *EgCCR *promoters

To investigate the ability of MYB sites of *EgCAD2 *and *EgCCR *promoters to interact *in vitro *and *in vivo *with MYB factor, site-directed mutagenesis was performed to disrupt these putative regulatory sites individually or in combination. Electrophoretic mobility gel assays (EMSA) and transactivation experiments were carried out in the presence of the *Eucalyptus *protein *Eg*MYB2, a R2R3 MYB factor, that was hypothesized to positively regulate the transcription of genes belonging to the monolignol pathway [23].

**Figure 3:**
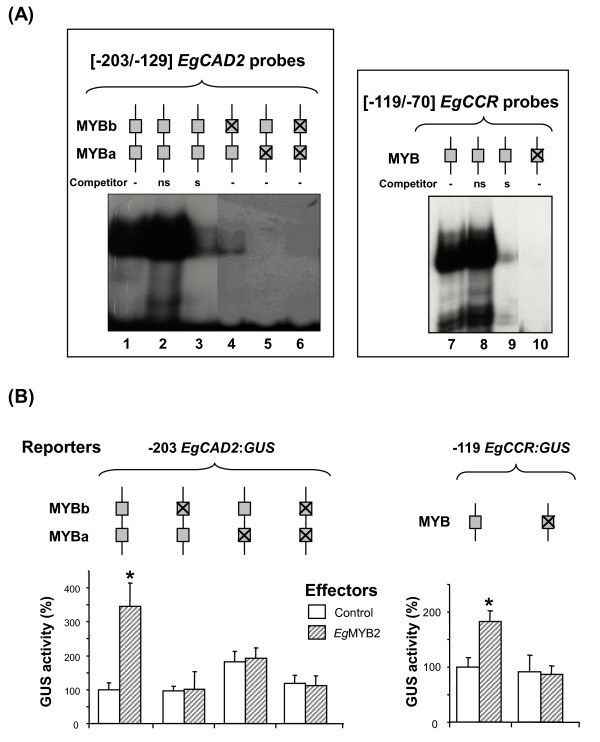
**Binding of *Eg*MYB2 to the regulatory regions of the *EgCAD2 *and *EgCCR *promoters containing mutated MYB element(s)**. **(A) **EMSAs were performed with recombinant *Eg*MYB2 protein (0.125 μg) and radiolabelled *EgCAD2 *[-203/-129] or *EgCCR *[-119/-70] fragments containing individual or multiple mutated MYB elements, as indicated above the lanes by crossed boxes. Lanes 2, 3, 8 and 9 each contain a 100-fold molar excess of unlabelled non-specific (ns, unrelated DNA fragment) or specific (s, *EgCAD2 *or *EgCCR *regulatory fragments, respectively) competitor fragment. (**B) **Transactivation assays were performed by co-transfecting reporter constructs (GUS fused to the -203 *EgCAD2 *or -119 *EgCCR *promoters containing the indicated mutated MYB elements) and effector constructs (*EgMYB2 *cDNA (*Eg*MYB2, hatched bars) or no cDNA (control, open bars) driven by the *CaMV 35S *promoter) in *Nicotiana benthamiana *leaves. GUS activity is expressed relative to that driven by the wild-type promoter controls. Histograms represent mean values and standard deviations of two independent experiments, each with three replicates. Activations of *EgCAD2 *and *EgCCR *promoters that are statistically significant relative to controls are highlighted by an asterisk (Student's *P *= 0.01 and 0.003, respectively).

EMSAs were performed using recombinant *Eg*MYB2 and DNA probes corresponding to [-203/-129] *EgCAD2 *and [-119/-70] *EgCCR *regulatory fragments with mutated MYB sites (Fig. 3a). The specificity of the DNA-protein complex was verified by competition with a 100-fold molar excess of unlabelled specific competitor (corresponding to the respective *EgCAD2 *and *EgCCR *regulatory fragments, lanes 3 and 9) whereas an unlabelled non-specific DNA (unrelated DNA fragment, lanes 2 and 8) did not affect the formation of the complexes. Mutation of the MYBa and MYBb sites in *EgCAD2 *and mutation of the MYB site in *EgCCR *abolished the binding of recombinant *Eg*MYB2 to the regulatory fragments. This loss of binding occurred even when a single MYB site remained intact in the *EgCAD2 *promoter, indicating that both MYB sites are required for maximal binding of *Eg*MYB2.

Transactivation experiments were performed by co-transfecting both the *EgMYB2 *cDNA expressed under the control of the *CaMV 35S *promoter (effector construct) and wild-type or mutated -203 *EgCAD2 *or -119 *EgCCR *promoters-*GUS *fusions (reporter constructs) into leaf mesophyll cells (Fig. 3b). When the wild-type -203 *EgCAD2 *promoter was co-transfected with the *EgMYB2 *construct, GUS activity was induced 3.4-fold when compared to the control construct (plasmid without *EgMYB2 *cDNA). Interestingly, the endogenous activity of the MYBa-mutated promoter was notably higher than that of the wild-type promoter, suggesting that the MYBa element might bind a repressor, at least in leaf mesophyll cells. Single or combined mutations of the MYBa and MYBb elements reduced the GUS expression driven by *Eg*MYB2 to the same level as their respective controls, thereby reflecting the loss of activation by *Eg*MYB2. Again, this effect was observed even with a single mutated MYB site, indicating that both MYB sites are required for transactivation of the *EgCAD2 *promoter by *Eg*MYB2. Transactivation of the -119 *EgCCR *wild-type promoter by *Eg*MYB2 by about 2-fold was also abolished upon mutation of the MYB site (Fig. 3b).

Together, these *in vitro *and *in vivo *data indicate that, in the experimental conditions used, the three MYB *cis*-elements are able to directly interact with *Eg*MYB2 transcription factor and are involved in the transcriptional activation of *EgCAD2 *and *EgCCR *genes mediated by *Eg*MYB2.

### Effect of mutations in *cis*-elements on the protein binding activities of the *EgCAD2 *and *EgCCR *regulatory regions

**Figure 4:**
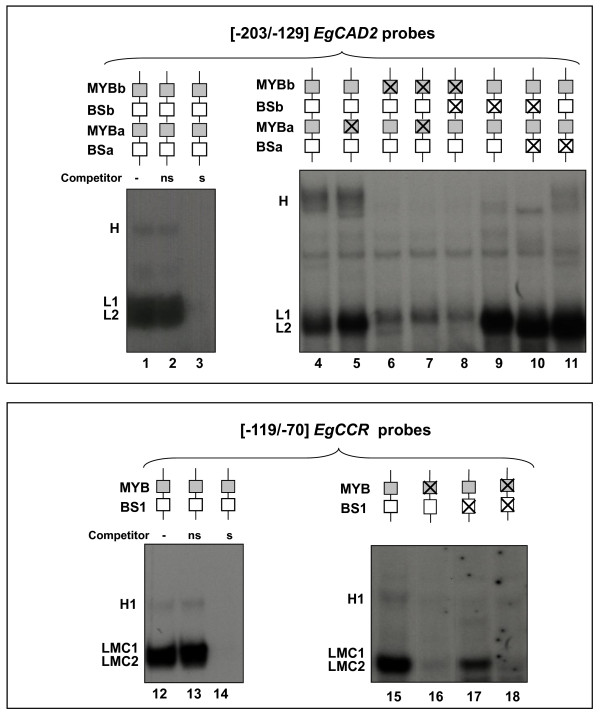
**Protein binding activities of the regulatory regions of *EgCAD2 *and *EgCCR *promoters containing mutated *cis*-element(s)**. EMSAs were performed with tobacco BY2 cell protein extracts (10 μg) and radiolabelled *EgCAD2 *[-203/-129] or *EgCCR *[-119/-70] fragments containing individual or multiple mutated *cis*-elements, as indicated above the lanes by crossed boxes. Lanes 2, 3, 13 and 14 each contain a 100-fold molar excess of unlabelled non-specific (ns) or specific (s) competitor fragment. Complexes are labelled H for high and L for low (see text).

To investigate the role of MYB and BS putative *cis*-elements of *EgCAD2 *and *EgCCR *promoters in the binding of transcription factors to the regulatory regions, EMSAs were performed using the regulatory fragments carrying several combinations of block mutations and tobacco protein extracts (Fig. 4). For both wild-type promoter fragments, a large fast-migrating band was observed and another slower-migrating complex (called H or H1, for higher) was also detected when the protein extracts were used immediately after extraction (Fig. 4, lanes 1, 4 and 12, 15). The large band was composed of two closely migrating complexes already described for *EgCCR *(LMC1 and LMC2, [5]) and named L1 and L2 for *EgCAD2*.

Competition with a 100-fold molar excess of the corresponding unlabelled specific competitor DNA fragments blocked the formation of all the complexes (Fig. 4, lanes 3 and 14) whereas a similar amount of an unlabelled non-specific DNA had no effect (Fig. 4, lanes 2 and 13), indicating the specificity of the interactions.

Whereas mutation of the MYBa site in *EgCAD2 *had no major effect on the EMSA result (Fig. 4, lane 5), mutation of the MYBb site prevented the formation of both the slower migrating complex (H) and the lowest part of the faster migrating large band (L2) (Fig. 4, lane 6). Single or double mutations in the BS elements of *EgCAD2 *somewhat perturbed the pattern of the complexes with a notable decrease in H and a concomitant increase in L2 (Fig. 4, lanes 9-11), especially when BSb was mutated (Fig. 4, lanes 9, 10). This concomitant increase in L2 was lost with the additional mutation in MYBb (Fig 4, lane 8). Thus, when combined mutations including MYBb were tested (*i.e*. MYBa + MYBb, lane 7 or BSb + MYBb, lane 8), similar results were obtained as with the mutation of MYBb alone, *ie *loss of the H and L2 complexes. Taken together, these data suggest that MYBb is a key *cis*-element involved in the formation of the H and L2 complexes and that BSb, and to a lesser extent BSa, apparently influence the ratio between the H and L2 complexes. Concerning the *EgCCR *promoter, only the mutation of the MYB element led to an overall decrease in all the complexes (Fig. 4, lanes 16, 18).

### Effect of mutations in *cis*-elements on vascular expression of *EgCAD2 *and *EgCCR *promoters

To investigate the role of each *cis*-element on tissue-specific expression from *EgCAD2 *and *EgCCR *promoters, transgenic tobacco plants expressing *GUS *under the control of the wild-type or mutated -203 *EgCAD2 *or -119 bp *EgCCR *promoters were generated.

**Figure 5:**
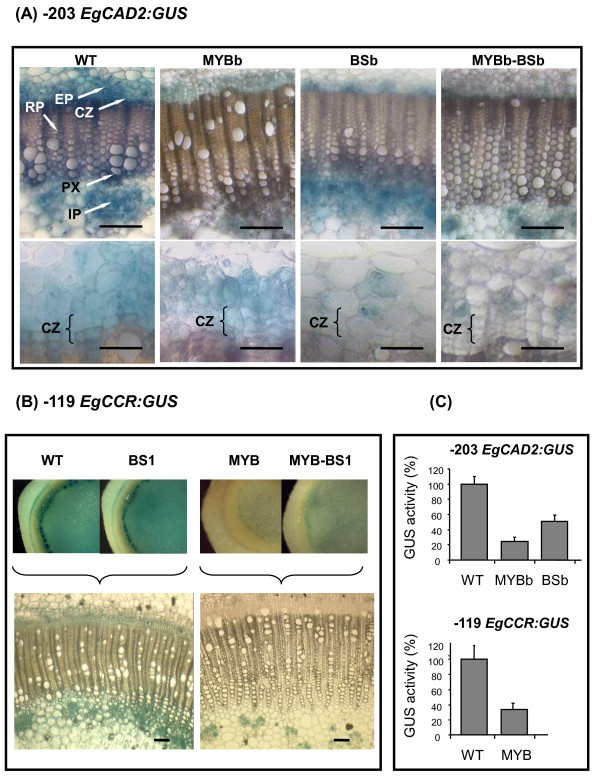
**GUS expression driven by *EgCAD2 *and *EgCCR *promoters containing mutated *cis*-elements**. **(A, B) **Histochemical analyses of GUS activity, driven by the wild-type or the -203 *EgCAD2 *and -119 *EgCCR *promoters mutated in the indicated *cis*-elements, in stem cross-sections of 8-week-old transgenic tobacco plants. **(A) **Upper panels, general views of the vascular tissues; lower panels, enlargements of the cambial zone, bars represent 200 μm and 50 μm, respectively. CZ, cambial zone; EP, external phloem; IP, internal phloem; PX, primary xylem; RP, xylem ray parenchyma.** (B) **Upper panels, general views of stem cross-sections; lower panels, enlargements of the vascular tissues. Bars represent 200 μm. (**C) **Fluorimetric assays of GUS activity in stems. Histograms represent mean values and standard deviations, expressed as % of the respective wild-type promoter-GUS activities. 7-10 independent transformants were examined for each construct. Activities driven by mutated *EgCAD2 *and *EgCCR *promoter are statistically different relative to the control (*WT -203 EgCAD2 or WT -119 EgCCR)*, for MYBb, BSb and MYB Student's *P *= 9 10-7, 9 10-4 and 0.06, respectively.

The BSa- or MYBa-mutated *EgCAD2 *promoters (see additional file 3) drove a GUS expression pattern similar to the wild-type -203 *EgCAD2 *promoter (Fig. 5a). In contrast, mutation of MYBb resulted in a considerable global diminution of GUS staining in all vascular tissues, suggesting that MYBb could be bound by an activator factor (Fig. 5a). This decrease in GUS staining was particularly pronounced in the xylem rays and in the parenchyma cells surrounding the primary xylem. Although with a lower intensity, the cambial-phloem zone still appeared as a continuous ring like with the wild-type promoter (Fig. 5a). This GUS expression pattern was also observed with the MYBa-MYBb double mutant (see additional file 3), confirming that MYBa does not play a crucial role in the promoter activity under these experimental conditions.

Upon mutation of the BSb *cis*-element, a substantial decrease in GUS activity was observed in the vascular cambium and, to a lesser extent, in the immediately derived parenchymatous cells, *ie *the xylem rays on the inner side and the parenchyma cells between phloem islands on the outer side (Fig. 5a). Thus, the cambial-phloem zone resulted in a spotted pattern with the spots showing GUS activity restricted to the external phloem area. The internal phloem still displayed GUS staining (Fig. 5a).

The double mutation BSb-MYBb reflected the combined actions of the single mutations with an overall reduction of GUS activity.

GUS activity driven by the wild-type -119 *EgCCR *promoter construct showed a vascular pattern similar to that driven by the -203 *EgCAD2 *promoter and to that reported in [5] using the full-length *EgCCR *promoter (Fig. 5b). This staining pattern was not affected by mutation of the BS1 *cis*-element. In contrast, GUS activity in plants carrying the single MYB mutant construct or the BS1-MYB double mutant construct notably diminished in all vascular tissues. As already observed for *EgCAD2*, GUS expression remained evident in the internal phloem of stem (Fig. 5b).

These observations were further corroborated by fluorimetric measurement of GUS activities (Fig. 5c). *EgCAD2 *MYBb and BSb single mutants showed a strong reduction of GUS activity (76% and 50%, respectively) when compared to the wild-type -203 promoter. Similarly, mutation of the MYB site in the *EgCCR *promoter resulted in a marked decrease of GUS activity (63%) when compared to the wild-type -119 promoter (Fig. 5c).

Taken together, these data provide evidence for a functional module comprising BSb-MYBb in the *EgCAD2 *promoter: MYBb appears to bind a general activator factor in all cell types whereas BSb might bind a protein specific to the cambium dividing cells. In the *EgCCR *promoter, the unique MYB *cis*-element might fulfil these dual functions.

## Discussion

### Contribution of the *EgCAD2 *and *EgCCR cis*-elements to the protein binding and to transcriptional activities of the promoters

Here, we delineated a short regulatory region [-203/-129] in the *EgCAD2 *promoter which is required for expression both in xylem tissues and in dividing undifferentiated cells either cultivated *in vitro *or already present *in planta *embedded within other tissues such as the cambial cells. Four *cis*-elements potentially involved in the regulation of *EgCAD2 *expression were identified within this region. They are arranged in two modules, each comprising an identical repeat of an unreferenced protein binding site (BS) with the sequence CTGGTT and an MBSIIG consensus site for MYB proteins. A simpler organisation exists in the [-119/-70] regulatory region of the *EgCCR *promoter with two putative *cis*-elements: a G-rich box (BS1) and a MBSIIG MYB consensus site [5].

Our results with EMSAs and transgenic plants with mutated *cis*-elements revealed the importance of the MYBb *cis*-element in *EgCAD2 *and of the MYB *cis*-element in *EgCCR *for the formation of the high and low mobility complexes and for the transcriptional activation of the *EgCAD2 *and *EgCCR *promoters in vascular tissues.

It is intriguing that neither EMSAs nor the transgenic plant approaches provided clear evidence of a functional role for MYBa in *EgCAD2 *promoter activity. This does not exclude its participation in other promoter functions, such as the response to external stimuli [6], or mechanisms specific to woody plant species [4,6], or expression in other parts of the plant, as does, for example, the ACIII/MYB element of the bean PAL2 promoter, which drives expression specifically in flowers [19]. Moreover, our observation in transactivation assays that the endogenous activity of the MYBa-mutated promoter in the absence of *Eg*MYB2 was notably higher than the wild-type promoter activity, suggests that the MYBa element might bind a repressor, at least in leaf mesophyll, a tissue where *EgCAD2 *and *EgCCR *promoters are poorly expressed. Such repressors of lignin biosynthesis genes have already been demonstrated in the MYB family; some of them have broad tissue expression patterns whereas others are tissue-specific or are preferentially expressed in particular tissues [24-27].

Mutation of the BSa site in the *EgCAD2 *promoter or of the G-rich BS1 site in the *EgCCR *promoter had no major effect on complex formation *in vitro *or on promoter activity in transgenic plants. In contrast, mutation of BSb in *EgCAD2 *drastically reduced the formation of the H complex in EMSAs and altered the expression pattern in transgenic plants, leading to decreased promoter activity in cambial cells and in the immediately derived parenchyma cells.

Ongoing studies show that the proteins of the slow-migrating H complex are found in the phosphorylated fraction of tobacco nucleus proteins whereas those of the faster-migrating L2 complex are found in the unphosphorylated one (data not shown). We believe that the H and L2 complexes which both bind the MYBb element of *EgCAD2 *could differ from each other by the addition of one or more protein(s) which can be subjected to phosphorylation. BSb that is located close to MYBb in *EgCAD2 *could be involved in the formation of the phosphorylated complexes since its disruption affects the ratio between H and L2. The possible involvement of the BSb site both in phosphorylated transcriptional complexes and in *EgCAD2 *expression in cambial cells (dividing cells) is consistent with the crucial and widely known role of phosphorylation mechanisms in metabolism connected to cell division [28].

The similar protein binding activities of the *EgCAD2 *and *EgCCR *regulatory fragments in EMSAs and the similar expression patterns driven by the promoters in vascular tissues suggest that the transcriptional mechanisms necessary for their coordinated expression may be mediated by their common MYB *cis*-elements.

### Functional architecture of the promoters

The complex organisation of the *EgCAD2 *promoter with its *cis*-elements arranged in two similar modules (BS-MYB) suggests that redundancy and mechanisms of cooperation or competition between *cis*-elements and *trans*-acting factors might be involved in the regulation of the promoter activity. In particular, EMSAs with recombinant *Eg*MYB2 and transactivation assays clearly demonstrated that the two MYB sites of the *EgCAD2 *promoter cooperated for binding and activation. When studying *in silico *the distribution of MYB sites in the proximal 500 bp of the *Arabidopsis *phenylpropanoid gene promoters (core, monolignol and flavonoid pathways), it appears that two copies of MYB elements and, more specifically, MBSIIG or MBSII sites (as defined by [22]) are found in most promoters, generally separated by 50 to 100 bp (see additional file 4, [15,16]). Although the *CCR *gene involved in lignin biosynthesis in *Arabidopsis *follows this rule, intriguingly the promoter of the *CCR *gene in *Eucalyptus *contains a single MYB element (see additional file 4). The two nearby MBSIIG of bean PAL2 and PAL3 genes (core phenylpropanoid pathway) were demonstrated to be crucial for the transcriptional regulation of these genes *in planta *[19,29], suggesting a functional significance for this promoter architecture. Furthermore, as in our study, cooperative binding was also reported for the two MYB elements of the bean PAL2 promoter in the presence of the pine *Pt*MYB1 protein [30].

Combinatorial interactions between the transcription factors binding to MYB elements and those binding to other closely located *cis*-elements are crucial for transcriptional regulation of the flavonoid branch pathway genes [31-33]. Other putative *cis*-elements that could cooperate with MYB elements were detected in the regulatory regions of *EgCAD2 *and *EgCCR *by using the PLACE database (http://www.dna.affrc.go.jp/htdocs/PLACE/signalscan.html, [34]). GT-1 elements, putative targets for trihelix-related transcription factors [35], appear to be well conserved in the regulatory regions of *CAD2 *and *CCR *in various *Eucalyptus *species (see additional file 2). Interestingly, the hypersensitive site at -187 revealed in this study by *in vivo *footprinting falls within a WRKY transcription factor binding site [36] that is conserved in the *CAD2 *regulatory regions; however it is more variable in *CCR*, especially in the invariant core which is essential for WRKY protein binding. In addition, the *CAD2 *regulatory region contains a conserved motif similar to the TERE *cis*-element that is involved in secondary wall formation during differentiation of tracheary elements [37]; this motif is, however, not found in the *EgCCR *regulatory region. Presently, we cannot exclude the involvement of these putative *cis*-elements in *CAD *or *CCR *expression. However, the strong conservation of the MYB elements together with their key role in promoter activity, as demonstrated in this paper, indicates that the coordinated developmental regulation of *EgCAD2 *and *EgCCR *expression is mediated through MYB transcription factors.

### MYB candidates for transcriptional regulation of *EgCAD2 *and *EgCCR *genes

*EgCAD2 *and *EgCCR *genes which, as demonstrated in this study, required functional MYB binding sites in their promoters, exhibit expression in cell types consistent with their function in monolignol biosynthesis. Their expression was indeed found (i) in xylem tissues undergoing lignification and in parenchyma cells surrounding and feeding the lignified tissues with monolignols and (ii) in the vascular cambium in which small monolignol oligomers may be components of a signal transduction pathway leading to the cell division [38].

MYB transcription factors responsible for *EgCAD2 *and *EgCCR *transcriptional activation should be found in the cell types exhibiting *CAD2 *and *CCR *expression and the interplay between various MYB factors or other transcription factors might contribute to the spatio-temporal expression patterns of *EgCAD2 *and *EgCCR *genes within specific cell types.

Particular MYB transcription factors have been detected in the cambium [39] and in xylem rays [40], and might contribute to *EgCAD2 *and *EgCCR *promoter activities in such parenchymatous tissues. Other MYB factors appear more specifically expressed in relation to secondary wall formation in cells that synthesize lignins. The *Eucalyptus Eg*MYB2 and the pine (*Pinus taeda*) *Pt*MYB1 and *Pt*MYB4 transcription factors are preferentially expressed in secondary xylem. When overexpressed, these transcription factors affect the expression of monolignol biosynthesis genes and the amount or composition of lignins [23,41,42]. In *Arabidopsis*, the transcriptional network regulating secondary wall synthesis has been investigated [43]. *At*MYB46, a close homologue of *Eg*MYB2 and *Pt*MYB4, appears to be a key switch in mediating the biosynthesis of the three major components of secondary walls (*i.e*. cellulose, hemicellulose/xylan and lignin) through the control of downstream, pathway-specific MYB and other transcription factors [44]. Recently, other *Arabidopsis *MYB factors, more closely related to *Pt*MYB1, were shown to directly regulate the promoters of lignin biosynthesis genes [43,45]. At the present time, the role of *Eg*MYB2 in the regulation of *EgCAD2 *and *EgCCR *gene expression must be clarified in order to determine whether *Eg*MYB2 activates the transcription of these genes by direct binding on their promoters *in vivo *or by an upstream control as suggested for *At*MYB46.

A complex picture of the transcriptional regulatory network controlling lignin biosynthesis genes is therefore emerging, with various MYB factors acting at different levels. As in the flavonoid branch pathway [31], the MYB factors controlling the monolignol pathway are likely to cooperate within multiprotein complexes with other protein partners that remain to be characterised.

## Conclusions

The overall goal of this project was to characterize the functional role of putative *cis*-elements from promoters of genes involved in the monolignol biosynthetic pathway. In this study, we first demonstrated that the MYB *cis*-elements found in the delineated regulatory regions of *EgCAD2 *and *EgCCR *promoters are able to be bound by MYB factors to form specific DNA-protein complexes *in vitro*. MYB *cis*-elements play a key role in the vascular expression of these two genes and support the hypothesis that MYB elements serve as a common means for the coordinated regulation of genes in the entire lignin biosynthetic pathway. Moreover, a novel functional *cis*-element was identified and shown to be involved, possibly in combination with the MYB *cis*-element, in the transcriptional regulation of *EgCAD2 *in cambial cells. A comprehensive understanding of the functional *cis*-elements involved in the transcriptional regulation of *EgCAD2 *and *EgCCR *is the first step to dissect the regulatory network controlling lignin biosynthesis. Future studies will aim at the identification of the transcription factors that bind these *cis*-elements and at the mechanisms by which these transcription factors interplay with each other to provide the final spatio-temporal regulation of the lignin pathway.

## Methods

### Plant material and transformation

Tobacco plants (*Nicotiana tabacum *cv. Samsun NN) were grown *in vitro *and in the greenhouse as described previously [46]. Tobacco leaf disk transformation with *Agrobacterium *was performed as described by [6]. Tobacco cells (*Nicotiana tabacum *cv Bright Yellow 2, BY2) were grown at 25°C and transformed with *Agrobacterium *as described by [47]. *Eucalyptus *cell suspensions were grown as in [48].

### Nucleic acids methods

Recombinant DNA methods were as recommended in [49]. DNA sequencing was performed with an ABI Prism 3700 DNA sequencer, using the ABI PRISM Dye terminator Cycle Sequencing Ready Reaction Kit (Applied Biosystems). The gene-specific primers used in this work and referred to hereafter are reported in additional file 5.

### *In vivo *footprinting

*In vivo *DMS treatment of *Eucalyptus *suspension-cultured cells was performed as described in [50]. Briefly, cells were treated with 0.5% dimethylsulfate (DMS) for 2 min and the reaction was stopped by 10-fold dilution with ice-cold water. As a control, genomic DNA extracted from *Eucalyptus *cells was treated *in vitro *under the same conditions. Methylated DNA was extracted using the DNeasy Plant Mini Kit (Qiagen) according to the manufacturer's instructions. *In vivo *and *in vitro *methylated DNAs were then cleaved with 1 M piperidine and recovered by lyophilisation and ethanol precipitation.

Methylated and cleaved DNA was used for ligation-mediated PCR (LMPCR) as described in [50]. Double-stranded, blunt-ended molecules were generated by primer extension from the *EgCAD2*-specific oligonucleotide 1 (see additional file 5). Ligation of the unidirectional common linker was performed as originally described by [21]. PCR amplification (15 cycles) was then performed by using DynazymeII DNA polymerase (Finnzymes) with *EgCAD2 *oligonucleotide 2 and common linker (additional file 5). Following amplification, *EgCAD2*-specific PCR products were labelled by extension from an *EgCAD2*-specific ^32^P-end-labelled oligonucleotide 3 (additional file 5). DNA was ethanol-precipitated and electrophoresed on a 6% sequencing polyacrylamide gel. The gels were dried and autoradiographed.

### Generation of *EgCAD2 *and *EgCCR *promoter-GUS fusion constructs

5'-deletions in the *EgCAD2 *promoter were obtained by PCR amplification of the pOGUS-*EgCAD2 *plasmid containing the full-length 2.5 kb *EgCAD2 *promoter [51] with primers that introduced an EcoRI site at positions -301, -247, -203 and a NcoI site at the ATG start codon (additional file 5). After re-cloning the PCR fragments into the pOGUS vector, the promoter-GUS cassettes were released by EcoRI and PstI digestion, and cloned into the pBluescriptSKM13+ vector to generate a XbaI site necessary for cloning into the pBin19 binary plasmid.

Site-directed mutagenesis was performed using the QuikChange Site-Directed Mutagenesis Kit (Stratagene) on *EgCAD2 *or *EgCCR *promoters in the pOGUS vector [5,51]. Two single-stranded complementary mutagenic oligonucleotides were used for each mutation (additional file 5). Double mutants were obtained from single mutants by using the same primers as for the single mutations. The PCR reactions were performed in 25 μl with 25 ng plasmid template, 0.2 mM dNTP, 1 unit Pfu Turbo DNA polymerase (Stratagene) and 62.5 ng of each mutagenic oligonucleotide. The parental strand was removed by digestion with DpnI. Mutated plasmid DNA was introduced into *E. coli *by electroporation. EcoR1-NcoI fragments were isolated by enzymatic digestion, purified by using the GenEluteä Gel Extraction Kit (Sigma) following the supplier's instructions, and fused to the *uidA *gene (GUS) in the pCambia1391Z binary plasmid (Genbank AF234312.1, Cambia, Canberra, Australia). Mutated constructs were introduced into *Agrobacterium tumefaciens *strain LBA4404 by electroporation. Promoter sequences and mutations were verified after each step by DNA sequencing.

### Electrophoretic mobility shift assay (EMSA)

The [-203/-129] *EgCAD2 *and [-119/-70] *EgCCR *promoter regulatory fragments used as probes for EMSA were obtained by amplification of pCambiaZ containing the wild-type or mutated *cis-*elements. Primers (4 pmol) common to the wild-type or mutated promoters were phosphorylated with 4 pmol of g[ ^32^P]-ATP (6000 Ci/mmol; Amersham) and T4 polynucleotide kinase (Promega) in 8 μl at 30°C for 30 min. The regulatory fragments were amplified by Pfu DNA polymerase with the radiolabelled common primer and variable second primers, depending on the introduced mutation (additional file 5). The labelled fragments were purified on a 4.8% polyacrylamide gel and eluted in water (100 μl) overnight at 4°C. The radioactivity incorporated was measured by Cerenkov counting.

Total protein extracts were prepared from exponentially growing 4-day-old tobacco BY2 cells by grinding in extraction buffer (50 mM Tris-HCl, pH 8, 500 mM NaCl, 10 mM MgCl, 10% glycerol, 7 mM β-mercaptoethanol, 1 mM EDTA, 0.5 mM PMSF). The BY2 cell protein extract was clarified by centrifugation for 30 min at 35000 g.

Binding reactions were performed at room temperature for 30 min in 25 μl binding buffer containing 25 mM Tris-HCl, pH 8, 250 mM NaCl, 10% glycerol, 7 mM β-mercaptoethanol, 2 μg poly (dIdC): poly(dIdC), 75 000 cpm of labelled probe and protein extracts. Free and bound DNAs were separated on 4.8% polyacrylamide gels in 0.5× TBE, 2.5% glycerol. Gels were then fixed, dried and autoradiographed.

### GUS fluorimetric and histochemical tests

GUS fluorimetric tests were carried out as described previously by using 4-methylumbelliferyl-β-D-glucuronide as substrate [6]. Measurement was done with a fluorometer (BIO-TEK FL600 Microplate Fluorescence Reader). Protein concentrations were determined by the Bradford method (Bio-Rad).

Histochemical localization of GUS activity was performed as described by [6] after pre-fixation in 0.3% formaldehyde in 10 mM MES, pH5.6, 0.3 M mannitol. General views were acquired with a binocular microscope (Leica MZ16) equipped with a camera (DC500, Leica). Transverse sections (300 μm) were cut on a vibratome with vibrating blade Leica VT1000S, mounted on glass slides and observed under an inverted microscope (Leitz DMIRBE, Leica Microsystems, Wetzlar, Germany). Images were acquired using a CCD camera (Color Coolview, Photonic Science, Millham, UK).

### Expression of GST*-Eg*MYB2 in *E. coli*

The *E. coli *strain BL21 containing the plasmid pGEX-5X-1-*EgMYB2 *cDNA was described in [23]. Induction of the GST-*Eg*MYB2 fusion protein was performed by adding isopropyl *β*-D-thiogalactoside (Sigma) to a final concentration of 0.1 mM. After growth at 28°C for 5 h, cells were lysed by two passages through a French press at 12000 p.s.i. (French^® ^Pressure Cell, Thermo Scientific, Waltham, MA, USA) in 20 mM potassium phosphate buffer, pH 7.5, 140 mM NaCl, 0.5 mM PMSF and 10 mM β-mercaptoethanol. The fusion protein was purified by FPLC (ÄKTA™ FPLC™ chromatographic system, Amersham Biosciences) on prepacked 1 ml GSTrap™ FF columns (Amersham biosciences) following the supplier's instructions. Proteins were eluted with 50 mM Tris-HCl, pH 8 containing 10 mM reduced glutathione protein (Sigma) and were then concentrated and dialysed by centrifugation on a Vivaspin concentrator (10000 MWCO, molecular weight cut-off, Vivascience) with 50 mM Tris-HCl, pH 8, 140 mM NaCl, 7 mM β-mercaptoethanol, 20% glycerol. Protein concentration was determined with the Bradford reagent (Bio-Rad).

### Co-transfection experiments

Co-transfection experiments were performed essentially according to the method of [52]. Effector constructs (pJR1 binary vector with or without *EgMYB2 *cDNA under the control of the cauliflower mosaic virus (*CaMV) 35S *promoter) were as in [23]. Reporter constructs (pCambiaZ binary vector containing wild-type or mutated *EgCAD2 *promoter-GUS or *EgCCR *promoter-GUS fusions) are described above. *Agrobacterium *strains GV3101:pMP90 containing effector or reporter constructs were co-infiltrated into leaves of *Nicotiana benthamiana *at the 5-leaf stage using a 1 ml syringe. After infiltration, the plants were maintained for 2 days in a growth chamber at 28°C under a photoperiod of 16 h light. Quantitative GUS assays were carried out on the infiltrated zone.

## Authors' contributions

AR and PR performed the experiments. AR helped to draft the manuscript. YM participated in the histological analysis. JGP initiated and coordinated the research. NCG and VP conceived and designed the study, supervised the assays, analyzed the data, discussed the results and wrote the paper. All authors read and approved the final manuscript.

## Supplementary Material

Additional file 1 ***EgCAD2 *gene expression in *Eucalyptus *suspension-cultured cells**. Click here for file

Additional file 2 **Sequence analysis of the regulatory regions of the *CAD2 *and *CCR *promoters from several *Eucalyptus *species**. Click here for file

Additional file 3 **GUS expression driven by *EgCAD2 *promoters containing mutated BSa, MYBa or MYBa-MYBb sites**. Click here for file

Additional file 4 **Position of MBSIIG and MBSII MYB consensus elements within the 500 bp proximal promoter regions of phenylpropanoid biosynthesis genes**. Click here for file

Additional file 5 **Oligonucleotide primers used for the various experiments**. Click here for file
