# Supplementary material for: Coordinated transcriptional regulation of two key genes in the lignin branch pathway - CAD and CCR - is mediated through MYB- binding sites
Source: BMC Plant Biol. 2010 Jun 28;10:130. doi: 10.1186/1471-2229-10-130 (PMC3017776; doi:10.1186/1471-2229-10-130)
Supplement: Additional file 1 — EgCAD2 gene expression in Eucalyptus suspension-cultured cells. [file 1471-2229-10-130-S1.PDF]

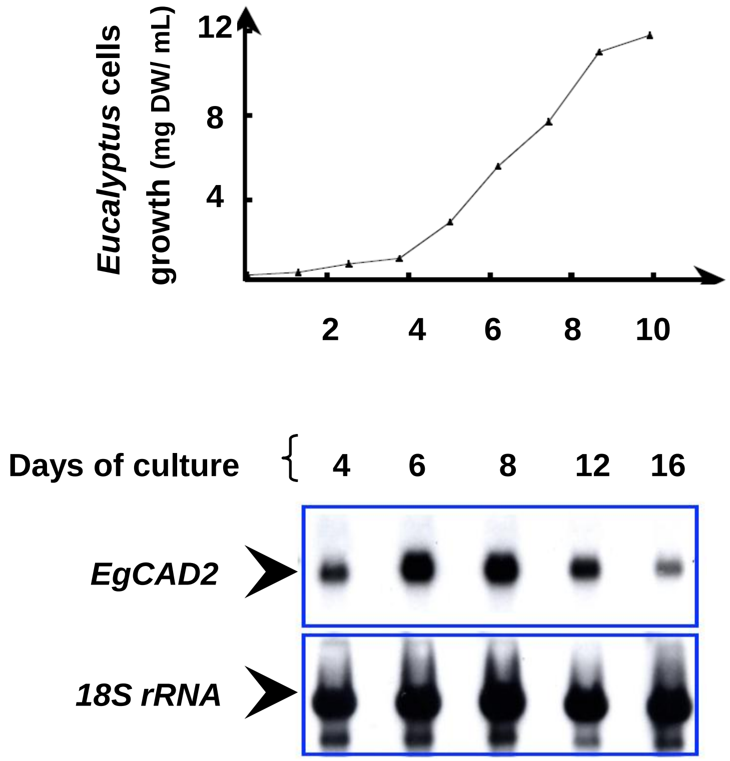

Additional file 1 - *EgCAD2* gene expression in *Eucalyptus* suspension-cultured cells. Total RNA (30 µg) was separated on glyoxal/DMSO agarose gels, transferred onto nylon membranes (Amersham) and hybridized to radiolabelled DNA probes: full-length *EgCAD2* cDNA or cDNA corresponding to the radish *18S* rRNA (kindly provided by Yves Meyer; CNRS, Perpignan, France).
