# Supplementary material for: Coordinated transcriptional regulation of two key genes in the lignin branch pathway - CAD and CCR - is mediated through MYB- binding sites
Source: BMC Plant Biol. 2010 Jun 28;10:130. doi: 10.1186/1471-2229-10-130 (PMC3017776; doi:10.1186/1471-2229-10-130)
Supplement: Additional file 2 — Sequence analysis of the regulatory regions of the CAD2 and CCR promoters from several Eucalyptus species. [file 1471-2229-10-130-S2.PDF]

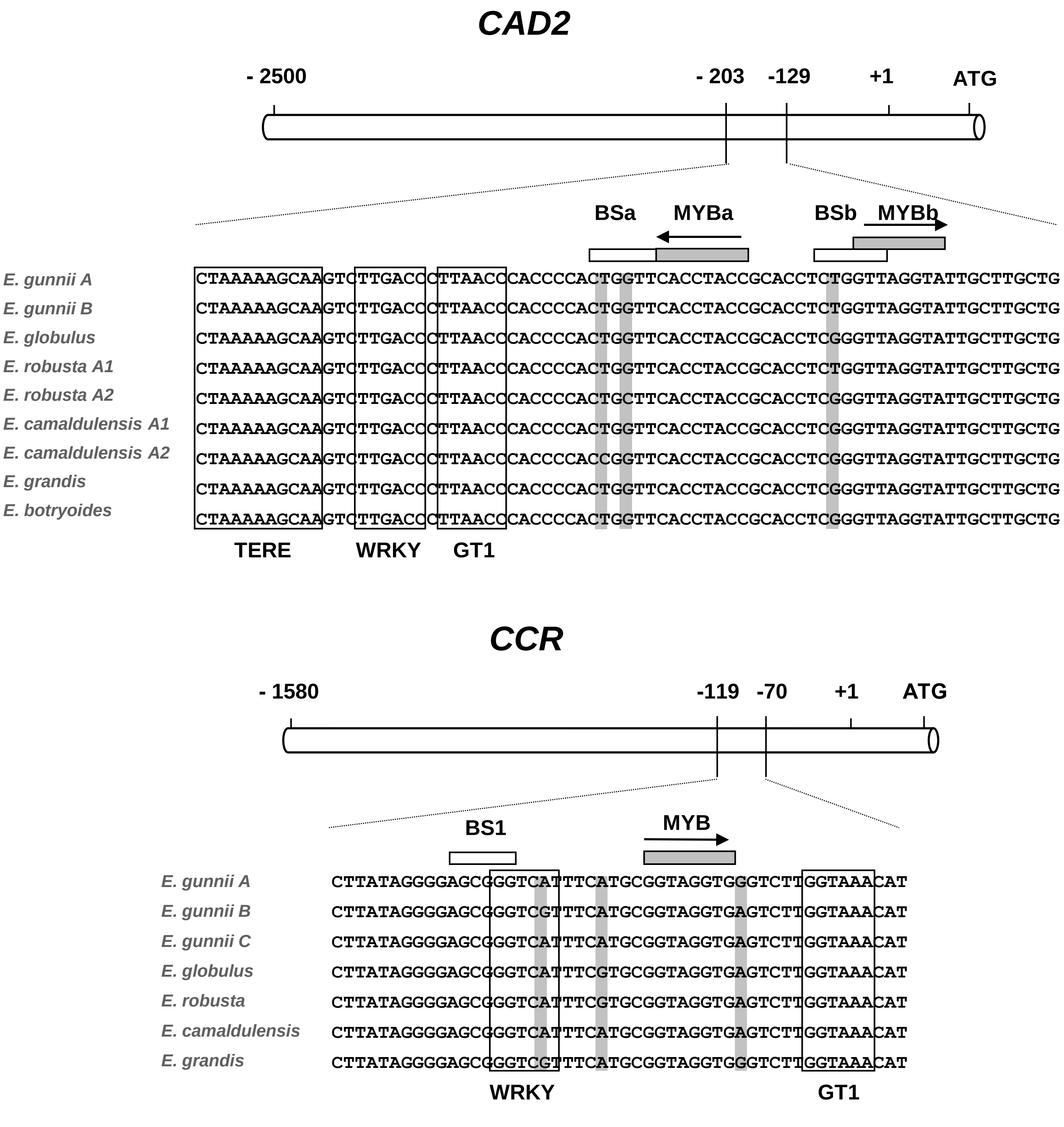

Additional file 2 – Sequence analysis of the regulatory regions of the *CAD2* and *CCR* promoters from several *Eucalyptus* species. Schematic maps of *CAD2* and *CCR* full-length promoters are indicated above the enlargements of the regulatory regions; (+1), transcription start site. Nucleotide sequences of the regulatory regions from the indicated species were obtained by PCR amplification based on *Eucalyptus gunnii* sequences (accession numbers: *EgCAD2*, GenBank X75480; *EgCCR*, GenBank X97433), except for the *E. botryoides CAD* sequence which was retrieved from the database (GenBank D16624). Sequences were obtained from different individuals of *E. gunnii* named A, B and C (in which A represents the original sequence from the database) and different alleles from an individual were designated A1, A2. The putative binding sites are shown as white (BS) and grey boxes (MYB). Variant bases are highlighted by grey columns. Other putative *cis*-elements are framed (see text).
