# Supplementary material for: Coordinated transcriptional regulation of two key genes in the lignin branch pathway - CAD and CCR - is mediated through MYB- binding sites
Source: BMC Plant Biol. 2010 Jun 28;10:130. doi: 10.1186/1471-2229-10-130 (PMC3017776; doi:10.1186/1471-2229-10-130)
Supplement: Additional file 3 — GUS expression driven by EgCAD2 promoters containing mutated BSa, MYBa or MYBa-MYBb sites. [file 1471-2229-10-130-S3.PDF]

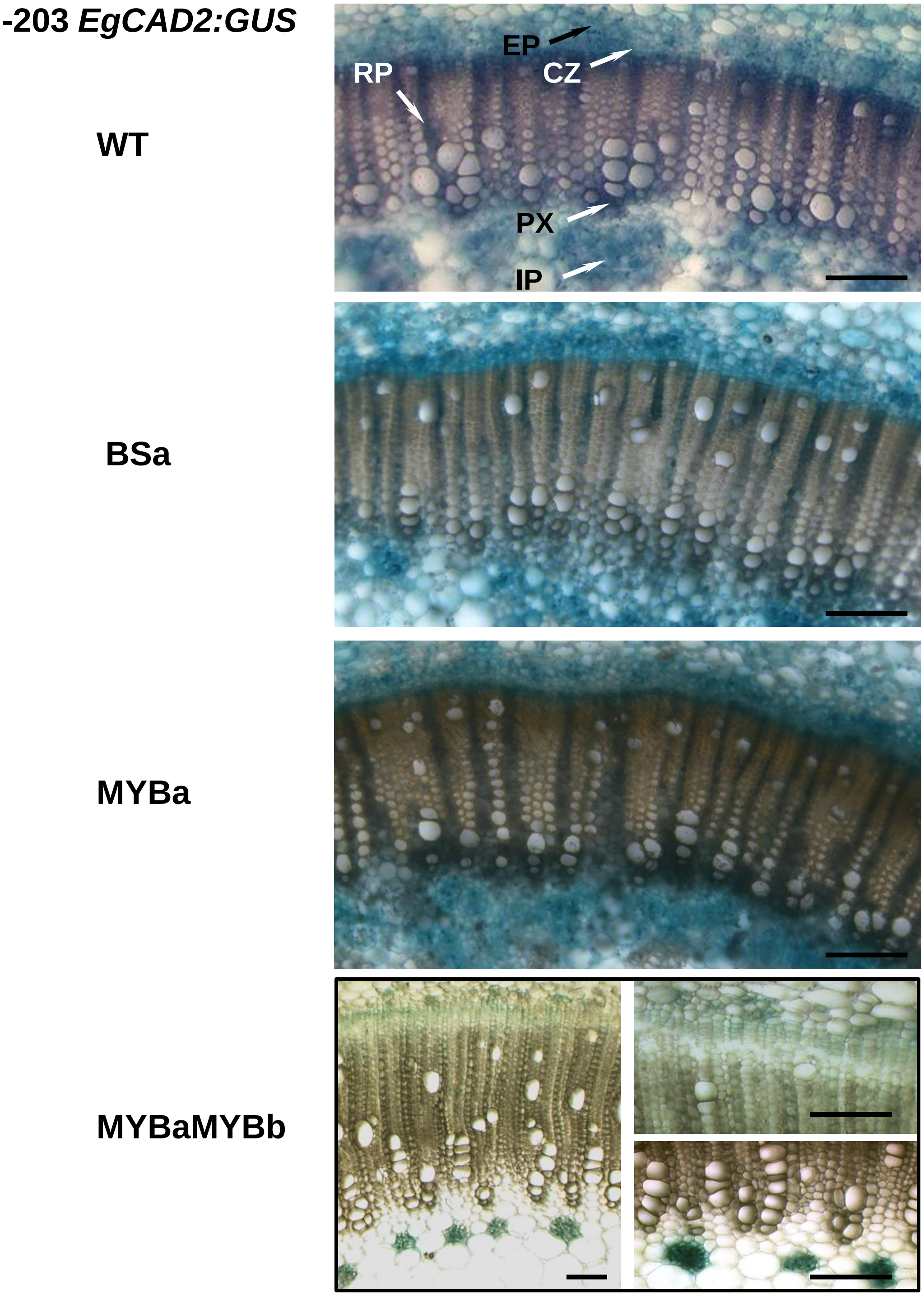

Additional file 3 – GUS expression driven by *EgCAD2* promoters containing mutated BSa, MYBa or MYBa-MYBb sites. Histochemical analyses of GUS activity, driven by the wild-type or the -203 *EgCAD2* promoters mutated in the indicated *cis*-elements, in stem cross-sections of 8-week-old transgenic tobacco plants. General views of the vascular tissues are shown for all plants. Additionally, enlargements of the cambial zone and of the internal phloem zone are shown for the mutated MYBa-MYBb construct. Bars represent 200 µm. CZ, cambial zone; EP, external phloem; IP, internal phloem; PX, primary xylem; RP, xylem ray parenchyma.
