# Supplementary material for: Coordinated transcriptional regulation of two key genes in the lignin branch pathway - CAD and CCR - is mediated through MYB- binding sites
Source: BMC Plant Biol. 2010 Jun 28;10:130. doi: 10.1186/1471-2229-10-130 (PMC3017776; doi:10.1186/1471-2229-10-130)
Supplement: Additional file 4 — Position of MBSIIG and MBSII MYB consensus elements within the 500 bp proximal promoter regions of phenylpropanoid biosynthesis genes. [file 1471-2229-10-130-S4.PDF]

Additional File 4 - Position of MBSIIG and MBSII MYB consensus elements within the 500 bp proximal promoter regions of phenylpropanoid biosynthesis genes

Consensus sequences for MYB elements as defined by Romero et al. (*Plant J* 1998, **14**:273-284) are shown at the top of the table in the forward and reverse orientations. The position of MYB elements in the listed genes is given in base pairs from the start of transcription (+1, as reported in the Plant Promoter Database, http://ppdb.gene.nagoya-u.ac.jp, Yamamoto and Obokata (*Nucleic Acids Res* 2008, **36**:D977-D981) and from the start of translation (ATG). Only one mismatch was allowed on the first or the last nucleotide of the consensus and is represented as a lower case character. The MYB elements that are strictly identical to the consensus are indicated in bold. The listed *A. thaliana* genes in the general phenylpropanoid pathway and in the monolignol-specific pathway are considered as the most probable genes to play a role in lignin biosynthesis (Goujon et al. *Plant Physiol Biochem* 2003, **41**:677-687).
(*), functionally tested MYB elements (this paper and Hartmann et al. *Plant Mol Biol* 2005, **57**:155-171)
#, overlapping MYB elements

Gene	Locus name	MBSIIG Consensus<br><br>YACCWAMC<br>GKTWGGTR	MBSII Consensus<br><br>YAACWAMC<br>GKTWGTTR	Position from +1	Position from ATG
EgCAD2	X75480	**-GTTAGGTA (*)**		**-139**	-255
		**-CACCTACC (*)**		**-156**	-272
		-TACCAAAa		-379	-495
EgCCR	AJ132750	**-GGTAGGTG (*)**		**-85**	-276
AtPAL1	At2g37040	**-TACCTACC #**		**-53**	-179
		**-CACCAACC**		**-348**	-474
AtPAL2	At3g53260	**-TACCTAAC #**		**-58**	-161
		-aACCAAAC		-259	-362
		-aACCAAAC #		-270	-373
			-aTTAGTTA	-364	-467
		**-CACCAACC**		**-383**	-486
			-CAACTACt	-453	-556
AtC4H	At2g30490	-aACCAAAC #		-46	-130
		**-TACCTAAC**		**-93**	-177
		-CACCAACt		-107	-191
			-CAACAACa	-180	-264
		**-TACCTACC**		**-193**	-277
		**-CACCAAAC**		**-230**	-314
At4CL1	At1g51680	**-CACCAACC**		**-61**	-150
		**-TACCAAAC**		**-161**	-250
			-CAACAAAg	-280	-369
At4CL2	At3g21240	**-CACCAACC**		**-45**	-115
			-aAACTACC	-88	-158
		**-CACCAACC**		**-154**	-224
		-tTTTGGTA		-488	-558
AtHCT	At5g48930	**-GGTAGGTG**		**-75**	-125
		**-TACCAACC**		**-177**	-227

			**-GGTTGTTG#**	**-363**	-413
AtC3H	At2g40890	**-CACCAACC**		**-41**	-136
		**-GTTTGGTG**		**-107**	-202
		-CACCAACt		-218	-313
AtCCoAOMT1	At4g34050	**-CACCAACC**		**-98**	-165
		-GTTTGGTc		-220	-287
			-CAACTACa	-441	-508
AtCOMT1	At5g54160		-tTTAGTTA	-276	-354
			-tGTTGTTA	-346	-424
		-tTTTGGTG		-459	-537
AtF5H1	At4g36220	-aACCTACC		-22	-83
		-TACCAAAa		-425	-486
			-GTTTGTTt	-452	-513
AtCCR1	At1g15950		-TAACTAAa	-1	-74
		**-GTTAGGTG**		**-77**	-150
			-GTTTGTTc	-141	-214
		**-CACCAACC**		**-187**	-260
		-tGTAGGTA		-288	-361
AtCAD4	At3g19450	-tTTTGGTA		-69	-167
		-CACCAAAt		-110	-208
		-GTTTGGTt #		-151	-249
		-tTTTGGTA		-197	-295
		**-TACCAAAC**		**-227**	-325
		-GGTTGGTt		-245	-343
		-CACCAAAa		-445	-543
AtCAD5	At4g34230	-CACCTAAa		-263	-316
			-tTTTGTTG	-288	-341
		**-CACCAACC**		**-453**	-506
			-GTTTGTTc	-460	-513
AtCHS	At5g13930		**-GTTTGTTA**	**-32**	-102
		-aACCTACC (*)		-68	-138
			-TAACAACt	-108	-178
			-tGTTGTTG #	-213	-283
			-cTTTGTTG	-382	-452
			-CAACAAAg	-392	-462
AtCFI	At3g55120	**-TACCTACC (*)**		**-40**	-95
AtF3H	At3g51240	**-GGTAGGTA (*)**		**-106**	-136
			**-GTTAGTTG**	**-158**	-188
		-TACCTACg (*)		-339	-369
AtFLS	At5g08640		**-GGTAGTTG (*)**	**-82**	-127
		**-TACCAAAC**		**-142**	-187
			-CAACAAAt	-193	-238
			-aAACTACC	-210	-255
			**-GTTTGTTG**	**-365**	-410
