# Supplementary material for: Coordinated transcriptional regulation of two key genes in the lignin branch pathway - CAD and CCR - is mediated through MYB- binding sites
Source: BMC Plant Biol. 2010 Jun 28;10:130. doi: 10.1186/1471-2229-10-130 (PMC3017776; doi:10.1186/1471-2229-10-130)
Supplement: Additional file 5 — Oligonucleotide primers used for the various experiments. [file 1471-2229-10-130-S5.PDF]

Additional File 5 - Oligonucleotide primers used for the various experiments

5' deletions of *EgCAD2* promoter

Forward primers (the EcoRI site corresponding to the 5' deletion is underlined):

EgCAD2 -301	5'-ATGGAATTCGTAAGAGGTCCACTGTACC-3'
EgCAD2 -247	5'-AAAACCAGAATTCCTGGAGGG-3'
EgCAD2 -203	5’-CAGAAACTGGGAATTCCTAAAAAGC-3’)

Reverse primer (the NcoI site encompassing the ATG codon is underlined):

EgCAD2, ATG	5’-GACTGCCCATGGTTGCTCAAAG-3’

***In vivo* footprinting of *EgCAD2* promoter** (reverse primers)

EgCAD2 oligonucleotide1, 5'-GGACTGCATATATATTTGGACAAAAACGC-3', position -15 to -43

EgCAD2 oligonucleotide 2, 5'-GGACAAAAACGCTACCAGCAACCAGCAACG-3', position -32 to -61

EgCAD2 oligonucleotide 3, 5'-CGCTACCAGCAACCAGCAACGAGGAAAGAGACGG-3', position -41 to -75

Mutation of *cis*-elements (only the forward strand of the two complementary oligonucleotides is shown for each mutation, the restriction sites introduced for the mutation are underlined)

EgCAD2, Bsa (HindIII)	5’-CCCACCCCAAGCTTTCACCTACCG-3’
EgCAD2, BSb (BamHI)	5’-CACCTACCGCAGGATCCGTTAGGTATTGC-3’
EgCAD2, MYBa (SalI)	5’-ACCCCACTGGTTCAGTCGACGCACCTC-3’
EgCAD2, MYBb (BglII)	5’-GCACCTCTGAGATCTTATTGCTTGCT-3’
EgCCR, BS1 (PstI)	5’- CCTTATAGGGGCTGCAGTCATTTCATG-3’
EgCCR, MYB (XbaI)	5’- CATTTCATGCGGTTCTAGAGTCTTGGTAA-3’

Sequencing of regulatory regions in various *Eucalyptus* species (primers were designed from *E. gunnii* sequences)

EgCAD2, forward	5’-AGGGAGTCAAGCATGTCAGAAGCA-3'
EgCAD2, reverse	5’- AAGCTTCTTTCAAAGTGGTGCTCGG -3’
EgCCR, forward	5’-CTCCAAGTACTAGAATCTCA-3’
EgCCR, reverse	5’- GCTTTTTCGGGCATGCAC -3’

Mutated promoter probes for EMSA

Common *EgCAD2* forward primer, 5'-TGGGAATGGCTAAAAAGCAA-3'
Specific *EgCAD2* reverse primer for wild-type MYBb, 5'-CTCAGCAAGCAATACCTA-3'
Specific *EgCAD2* reverse primer for mutated MYBb, 5'-CTCAGCAAGCAATAAGAT-3'
Common *EgCCR* reverse primer, 5'-GACAATGTTTACCAAGAC-3'
Specific *EgCCR* forward primer for wild-type BS1, 5'-CCTTATAGGGGAGCGGGTCAT-3'
Specific *EgCCR* forward primer for mutated BS1, 5’-CCTTATAGGGGCTGCAGTCATTTCATG-3’
